# Decoding Abilities in Adolescents with Intellectual Disabilities: The Contribution of Cognition, Language, and Home Literacy

**DOI:** 10.5334/joc.191

**Published:** 2021-10-01

**Authors:** Karin Nilsson, Henrik Danielsson, Åsa Elwér, David Messer, Lucy Henry, Stefan Samuelsson

**Affiliations:** 1 Linköping University, Sweden; 2 The Swedish Institute for Disability Research, Sweden; 3 The Open University, UK; 4 City, University of London, UK

**Keywords:** decoding, intellectual disabilities, RAN, phonological awareness

## Abstract

Decoding abilities in individuals with intellectual disabilities (ID) are substantially lower than for typical readers. The underlying mechanisms of their poor reading remain uncertain. The aim of this study was to investigate the concurrent predictors of decoding ability in 136 adolescents with non-specific ID, and to evaluate the results in relation to previous findings on typical readers. The study included a broad range of cognitive and language measures as predictors of decoding ability. A LASSO regression analysis identified phonological awareness and rapid automatized naming (RAN) as the most important predictors. The predictors explained 57.73% of the variance in decoding abilities. These variables are similar to the ones found in earlier research on typically developing children, hence supporting our hypothesis of a delayed rather than a different reading profile. These results lend some support to the use of interventions and reading instructions, originally developed for typically developing children, for children and adolescents with non-specific ID.

## Introduction

Students with intellectual disability (ID) have by definition low general intelligence (IQ below 70; American Psychiatric Association, 2013) and many exhibit severe difficulties with both decoding and reading comprehension (Lemons et al., 2013; Wei, Blackorby, & Schiller, 2011). Few studies target individuals with ID (Bishop, 2010), with the result that knowledge is limited about how, in students with ID, a combination of skills and environmental factors produce low levels of reading. Non-specific ID involves general cognitive impairments that concern conceptual, social and adaptive abilities, with an early developmental onset (American Psychiatric Association, 2013), but no specific diagnosis of another disability. Delays in many cognitive and language abilities are common (Danielsson, Henry, Messer, & Rönnberg, 2012; Danielsson, Henry, Rönnberg, & Nilsson, 2010; Henry, 2001; Henry & Winfield, 2010; van der Molen, Henry, & van Luit, 2014). Because IQ is neither a strong nor the best predictor of decoding abilities in typically developing children (Stanovich, 2005; Vellutino, Scanlon, & Lyon, 2000), the findings of severe difficulties with decoding in students with ID are somewhat surprising.

The present study focusses on adolescents with non-specific ID because there have been no previous systematic investigations of the variables contributing to decoding in this group. The key contribution of the present study is to include the broadest range of concurrent predictors of decoding (phonological decoding and word recognition), and one of the largest sample sizes of adolescents with ID, ever undertaken. Our purpose is to investigate which, and to what extent, individual abilities (cognition, language) and home literacy influence decoding abilities in students with ID. Our investigation of the variables contributing to decoding also involves an evaluation of delay and difference hypotheses about developmental disabilities. A delay hypothesis, as applied to our investigation, suggests that the variables related to decoding abilities in adolescents with ID will resemble the variables found in previous research on younger typically developing children. A difference hypothesis, when applied to this study, suggests that the variables related to decoding abilities in adolescents with ID will be different from the variables found in previous research on typically developing students.

This study was conducted on Swedish-speaking individuals with non-specific ID, enrolled in special schools. Swedish is a transparent orthography, which means that the patterns are consistent and learning to read proceeds more quickly in comparison to an opaque orthography, such as English (Patel, Snowling, & de Jong, 2004). The consistency of grapheme-phoneme correspondences in Swedish is comparable to other European languages, such as Dutch and German.

### Decoding abilities in typically developing students and those with dyslexia

A number of variables are known to predict decoding abilities, both concurrently and over time. Two review articles and two longitudinal studies have found similar predictors in research on typically developing students and students with reading disabilities, namely phonological awareness, letter-sound knowledge and rapid automatized naming (RAN; Hulme & Snowling, 2013; Landerl & Wimmer, 2008; Scarborough, 1998; Schatschneider, Fletcher, Francis, Carlson, & Foorman, 2004). In addition, a study by Kibby et al. (2015) revealed that visual sequential short-term memory was significantly related to decoding performance in students with reading disabilities, after controlling for phonological awareness and verbal intellectual ability. Other studies have found that individuals with developmental dyslexia have a reduced memory for visual and spatial information (Menghini, Carlesimo, Marotta, Finzi, & Vicari, 2010; Menghini, Finzi, Carlesimo, & Vicari, 2011). Verbal fluency, which is the ability to verbalize words rapidly starting with a specific letter or representing a specific category, has also been found to be impaired in students with dyslexia (M. J. Cohen, Morgan, Vaughn, Riccio, & Hall, 1999; Reiter, Tucha, & Lange, 2005; Smith-Spark, Henry, Messer, & Ziecik, 2017). The role of IQ in explaining individual differences in reading varies between reading tasks. Regarding decoding skills, most studies suggest only weak relationships with IQ (Stanovich, 2005; Vellutino, Fletcher, Snowling, & Scanlon, 2004), although, Tiu, Thompson, and Lewis (2003) found a significant relationship between IQ and measures of decoding in a group of participants with reading disabilities. Furthermore, executive-loaded working memory (ELWM; e.g. the ability to multi-task when processing and remembering information) is known to be related to IQ and is also associated with reading disabilities (Booth, Boyle, & Kelly, 2010).

Another area often assessed in research on reading concerns environmental factors. Segers, Damhuis, van de Sande, and Verhoeven (2016) found that parents’ educational level related to children’s decoding ability in first grade, and Noble, Farah, and McCandliss (2006) reported that socioeconomic background (a variable containing measures of parental education, occupation and income level) played an important role in predicting early reading development.

### Decoding abilities in students with ID

Many students with ID have severe difficulties on different types of reading tasks (Channell, Loveall, & Conners, 2013; Lemons et al., 2013). For example, Lemons et al. (2013) reported that 56% of students with ID at grade 11 exhibited a level of decoding that corresponded to grade 1 of typically developing children, and 14% passed the level corresponding to the average of grade 3. These findings showed seriously compromised decoding abilities in a large proportion of the students with ID. In another study, with students aged 6–21 years, one-third of the sample decoded words letter by letter (Ratz & Lenhard, 2013), which corresponds to the early phases of decoding in typically developing students (Ehri, 2005).

Despite these low levels of decoding abilities, the research on reading abilities in ID is sparse. Age and IQ are often used either as key effects or important matching criteria. The current literature on students with ID suggests that decoding is explained by: phonological awareness (Barker, Sevcik, Morris, & Romski, 2014; Channell, Loveall, & Conners, 2013; Saunders & DeFulio, 2007; Sermier Dessemontet & de Chambrier, 2015; Soltani & Roslan, 2013; van Wingerden, Segers, van Balkom, & Verhoeven, 2017); letter-sound knowledge (Sermier Dessemontet & de Chambrier, 2015; van Tilborg, Segers, van Balkom, & Verhoeven, 2014); and RAN (Barker, Sevcik, Morris, & Romski, 2014; Saunders & DeFulio, 2007; Soltani & Roslan, 2013; van Wingerden, Segers, van Balkom, & Verhoeven, 2017). In addition, a study by Conners, Atwell, Rosenquist, and Sligh (2001) showed that rehearsal in phonological memory was the only significant difference between stronger and weaker decoders while the significance of phonemic awareness disappeared when age was covaried out. This result differs from research on typically developing children, where phonemic awareness usually differentiates good and poor readers. Home literacy is seldom investigated in this group, although parents’ educational level was related to decoding ability in one study (Wei, Blackorby, & Schiller, 2011). In addition, differences in control variables that have been used, and the fact that studies focus on different groups with ID (e.g. Down syndrome, unknown aetiology) make comparisons between studies difficult.

One reason for the lack of research into reading and ID may be the assumption that the students’ low IQ explains their poor reading skills. However, intelligence is not considered a major predictor of decoding skills in typically developing children (Stanovich, 2005; Vellutino, Fletcher, Snowling, & Scanlon, 2004), and therefore it is important to identify the variables that contribute to decoding skills in students with ID. Without knowledge about the level of contribution of cognition, language, and home literacy, it is difficult to adapt teaching methods to support reading in students with developmental disabilities. It is important to note that the IQ discrepancy criterion is no longer used when diagnosing dyslexia and other learning disabilities (Stanovich, 2005), which means that some of our participants are likely to meet the modern criteria for a dyslexia diagnosis. However, our focus is on the aim of identifying predictor variables in the ID population, rather than whether or not a particular individual meets the criteria for dyslexia.

In summary, research on typically developing children in the early stages of reading has identified phonological awareness, RAN, and (mental) age as important variables for explaining variance in decoding skills. Consequently, if decoding involves delayed development in students with ID then these abilities should be the most important variables. If our findings indicate that other variables are important in explaining decoding skills this will support a difference hypothesis about decoding abilities in students with ID. Furthermore, the variables explaining decoding ability in students with ID have not been systematically evaluated before and existing knowledge is limited. As a result, the current study, with its large sample size and broad range of variables, will make an important contribution to the understanding of decoding abilities in students with ID. These findings are likely to be important for the development of teaching methods to support reading in this group.

### Present study

In the present study, 136 participants with non-specific ID between 12–19 years of age were assessed on the following variables: decoding (i.e. word recognition and phonological decoding of nonwords), phonological awareness, RAN, verbal fluency, grammatical understanding, receptive vocabulary, IQ, different aspects of working and short-term memory and home literacy. An innovative aspect to our investigation of decoding abilities was the assessment of the ability to process visual information, in particular visual memory abilities; because students with ID are a heterogeneous group, this was potentially a valuable addition. All variables included in our study were chosen on the basis that they have correlated with reading abilities in previous research. We chose not to include the variable letter-sound knowledge, because that measure is more reliable as a longitudinal predictor when assessed before children have started their formal reading instruction or in the early school years (Sermier Dessemontet & de Chambrier, 2015; van Tilborg, Segers, van Balkom, & Verhoeven, 2014).

### Research Question and Hypotheses

Our research question concerns how cognition, language, and home literacy influence decoding in students with ID. Two different hypotheses related to this questions were tested, namely a delay and difference hypothesis about decoding in students with ID. The delay hypothesis was supported if the most important variables contributing to decoding ability in adolescents with ID were the same as those found in previous research on younger typically developing children, namely RAN, phonological awareness, and (mental) age.

There is some evidence, although limited, that other variables might be important for decoding ability in students with ID, which would support a difference hypothesis. For example, Conners, Atwell, Rosenquist, and Sligh (2001) found that phonological short-term memory was significantly related to decoding, when the remaining variables were accounted for. Furthermore, research on individuals with reading disabilities has shown that this group has impairments in executive functions (Booth, Boyle, & Kelly, 2010) and in memory for visual and spatial information (Menghini, Carlesimo, Marotta, Finzi, & Vicari, 2010; Menghini, Finzi, Carlesimo, & Vicari, 2011). Tiu, Thompson, and Lewis (2003) also found a significant relationship between IQ and decoding measures in individuals with reading disabilities. These findings, therefore, point towards a difference hypothesis, whereby these other measures are found to be significantly related to decoding.

## Method

The data collection for this study was part of a larger data collection on reading ability in students with ID. The focus in the current study is on decoding ability, whereas reading comprehension will be addressed in another article (Nilsson et al., 2021).

### Power analysis

A power analysis was conducted with the pwr package (Champely, 2017) in R for a regression analysis with 13 variables, a medium effect size (f^2^ = 0.15) as provided in pwr based on J. Cohen (1988), alpha = 0.05, and power = 0.80, resulting in a minimum sample size of 131 participants. However, the partner study of reading comprehension (Nilsson et al., 2021) required a minimum sample size of 150, and consequently the plan was to include 150 participants in the present study. Therefore, the study would have 80% power to detect an effect size of Cohen’s f^2^ of 0.13.

### Participants

The planned inclusion criteria were: 1) age 12–19 years; 2) a level of decoding ability that could be measured with the tests used in this study; 3) normal or corrected to normal vision and hearing; 4) Swedish speaking home environment since birth; and 5) no developmental diagnoses other than non-specific ID. Comorbidities with other diagnoses, such as ADHD and ASD, are common in individuals with ID. As the recruitment of participants was too challenging (less than 50 participants recruited in 6 months of total recruitment/testing time), the fifth inclusion criterion was dropped. Due to financial constraints, the plan was to stop data collection after two years or 150 included participants, whichever happened first. After 6 months, only 17 participants were recruited. Hence, the fifth inclusion criterion was dropped, which resulted in 51% of the included participants being reported to have additional diagnoses. After two years, the data collection ended with 136 participants tested. However, the target of 150 participants would have been reached if the pandemic had not impacted testing of participants with consent the final 2 months of data collection. Participants were recruited via schools (upper secondary and high school) in Sweden. After initial contact from the research team, principals or teachers contacted students and parents. To be included in the study everyone involved (i.e. schools, parents, and adolescents) had to provide their consent. Participants and parents were initially asked to sign a letter of consent but all participants were also asked for oral assent before the assessment started.

We received a total of 176 consent letters, and 15 were excluded before testing due to the following reasons: presence of a syndrome (3); not speaking Swedish in home environment (6); not correct chronological age (2); and no name or contact information was included (4). In addition, 22 participants were not tested due to pandemic related school restrictions. Of the 139 tested participants, one was excluded because of inclusion criterion two (decoding was not tested), and two were excluded because of inclusion criterion three (not normal or corrected to normal hearing or hearing not tested). Our final sample consisted of 136 participants (59 girls). This sample size was considered large enough to proceed with our planned analysis. The mean chronological age was 189.61 months (*SD* = 25.87 months), the mean estimated IQ level of the participants was 59.43 (*SD* = 9.72), and the mean mental age was 112.88 months (*SD* = 25.26 months). More detailed information is provided in ***Table 1***. IQ level was estimated using two sub-tests from the WISC-V (Wechsler, 2014). Fifteen participants were estimated to have an IQ above 70, however, all participants were enrolled in special education classes during the data collection, which in Sweden means that they have been thoroughly tested and diagnosed as having ID and an IQ < 70 by a clinician.

**Table 1:** Descriptive statistics of participant characteristics and task performances (raw scores) of adolescents with intellectual disability (n = 136).


TEST	*M*	*SD*	MIN	MAX	SKEWNESS	KURTOSIS

Chronological age (months)	189.61	25.87	146	239	0.26	–1.02

Mental age (months)	112.88	25.26	63	190	0.62	0.31

IQ	59.43	9.72	40	88	0.30	–0.08

Word recognition timed	45.10	17.76	4	94	0.03	–0.55

Word recognition untimed	76.48	18.87	13	99	–1.02	0.32

Phonological decoding timed	23.28	11.75	2	55	0.32	–0.71

Phonological decoding untimed	36.18	16.38	2	61	–0.40	–1.02

Blending	15.71	3.66	2	20	–0.94	0.47

Elision	9.91	5.90	0	19	0.12	–1.47

46–items	18.26	14.93	0	43	0.26	–1.48

RAN colors	68.16	22.38	33	184	1.40	4.15

RAN letters	44.29	16.33	22	117	1.50	2.91

Verbal fluency category	25.87	9.35	4	58	0.50	0.73

Verbal fluency letters	19.01	9.95	0	48	0.57	–0.25

Vocabulary	131.15	27.35	33	179	–0.69	0.39

Grammatical comprehension	11.16	4.09	2	18	–0.40	–0.88

Phonological STM	8.42	2.57	3	18	0.56	1.11

Spatial STM	9.91	2.74	2	18	–0.16	0.32

Visual STM	9.62	4.13	0	16	–0.81	–0.23

Phonological ELWM	4.88	2.01	0	10	0.15	–0.40

Visuospatial ELWM	6.90	2.92	3	17	0.95	0.77

Home literacy	44.35	5.85	27	59	–0.16	–0.10


*Note*: Abbreviations: ELWM = executive-loaded working memory, STM = short-term memory.

### Assessment

All participants were assessed in their school environment on a range of cognitive and language skills. We examined the relationship between these skills and the dependent variable of decoding (a composite of word recognition and phonological decoding). Standardized measures were chosen where possible. All tests were administered in Swedish and all the translated tests had been used in previous research with Swedish participants. The tests for reading and language abilities had been used in a pilot study on the same population and turned out to work well. The research group previously had used the cognitive tests in assessing students with ID and all the tests were used successfully with this population. Assessments also included visual and auditory perception tests to rule out hearing and visual problems. The total testing time was estimated to be approximately 4 hours per participant, divided into sessions compliant with the school schedule. This estimation turned out to be correct. Sessions were completed during different days and breaks were allowed whenever necessary to avoid fatigue. Three test leaders (research assistant 1, months 1–14; research assistant 2, months 15–24; 1^st^ author months 1–24), who were formally trained in using all tests, conducted the assessments. All test leaders had prior experience of testing, and had trained to use the tests together. All data was recorded on paper. The data was entered by one test leader, and then re-entered by a second test leader to minimize errors. Tests measuring reading comprehension and listening comprehension, and a questionnaire about attitudes towards reading were also administered during the data collection. These variables are relevant to another article (Nilsson et al., 2021). The planned test order was: word recognition, IQ, vision, phonological decoding of nonwords, hearing, visual sequential memory, reading comprehension, verbal fluency, phonological awareness, RAN, listening span, vocabulary, listening comprehension, questionnaires, digit span, grammatical understanding, odd one out span, and the Corsi blocks test. Order alterations were allowed to take advantage of the whole testing session, such as moving time consuming tests to the next session. In order to minimize the risk of fatigue or the participants experiencing feelings of failure, nearly all the tests included stopping criteria. In some cases, where stopping criteria were not a part of the original test, they were added by the research team. In research on typically developing children, it is common to control for chronological age. However, from a developmental point of view it is more reasonable to use mental age for our sample, rather than chronological age or IQ.

#### Decoding

Decoding was measured using the test LäSt (Elwér, Fridolfsson, Samuelsson, & Wiklund, 2016). This test includes measurements of word recognition and phonological decoding. The test consists of two forms, A and B, covering both types of decoding. The A-form was used to assess timed decoding ability and the participants were instructed to read separate lists of words and nonwords as quickly as possible during 45 seconds. The B-form was used to assess untimed decoding ability and the participants were instructed to read the whole list of words and nonwords as accurately as possible. Testing with the B-form was stopped following 10 consecutive errors. The raw scores were calculated from the total number of correct words that were read on each form. The four measures of decoding were entered into a principal component analysis (PCA) and the main decoding variable used in the regression analysis was the PCA for a one component solution. As only one component was identified no additional regression analyses were conducted.

#### Receptive vocabulary

Receptive vocabulary was measured using the Peabody Picture Vocabulary Test, Third Edition (PPVT-III) (Dunn & Dunn, 1997). The participants were asked to match a stimulus word, which was presented orally by the examiner, to one of four black-and-white line drawings. The words included are nouns, adjectives and verbs and cover 20 content areas including animals, body parts, clothing, emotions, facial expressions and foods. The test is arranged in blocks of 12 items and has a total of 204 items. The blocks are arranged in order of increasing difficulty and testing is stopped following eight or more errors within one block. According to the manual, the level of difficulty of the initial testing should be related to the participant’s age. In this study, we started from the first block with all participants as it can be difficult to ascertain where to start graded tests in individuals with ID. Because the test is translated into Swedish, the order of increasing difficulty has been slightly changed which also justifies the decision to start with the easiest block. The raw score was the total number of correct answers.

#### Phonological awareness

Phonological awareness was measured using three different tests. Two of them were subtests from the Comprehensive Test of Phonological Processing (CTOPP) (Wagner, Torgesen, & Rashotte, 1999). The Blending Words subtest requires participants to blend sounds together to say a word. The examiner orally presents words pronounced in segments and the participants are asked to verbalize the whole word (e.g. What word do these sounds make /s/-/un/: sun). There are 20 items and testing was stopped following three consecutive errors. The Elision subtest requires the participants to repeat a word after the examiner and then say the word again but leaving certain sounds out (e.g. Say firetruck. Now say firetruck without saying truck: fire). There are 20 items and testing was stopped following three consecutive errors. The third test, called 46-items (Olson, Forsberg, Wise, & Rack, 1994), requires the participants to repeat nonwords presented orally by the examiner and then to say the word again but leaving certain sounds out, which makes the non-word a real word (e.g., “Say prot. Now say prot without the /r/ sound: pot”). There are 46 items and testing was stopped following five consecutive errors. Raw scores on each test in this section were the total number correct. All measures of phonological awareness were combined (sum of z-transformed measures) to give one variable that was used in the regression analysis.

#### Grammatical understanding

Grammatical understanding was measured using the Test for Reception of Grammar Version 2, TROG-2 (Bishop, 2003). The participants were instructed to select the correct picture corresponding to a phrase or sentence, presented orally by the examiner, from one of four coloured pictures. Items are divided into blocks of four and each block tests the understanding of a specific type of contrast (for example, reversible passives such as, “the girl is chased by the horse,” and embedded sentences such as, “the book the pencil is on is red”). The blocks are arranged in order of increasing difficulty. One block is considered correct when all four correct items are selected. Testing was stopped following 5 consecutive block errors. The raw score was the total number of blocks correct.

#### Verbal fluency

Verbal fluency was measured using the Delis-Kaplan Executive Function System sub-test (Delis, Kaplan, & Kramer, 2001), which involves several letter and category fluency tasks. The participants were asked to verbalize as many words as possible starting with a specific letter (three different letters were used: F, A, and S), and from two specific semantic categories (animals and boys’ names). Each task has a time limit of one minute. Words starting with a non-target letter, words that are not animals or boys’ names, and repetitions were counted as errors. The raw score was the total number of correctly generated words. Raw scores from both tasks were combined (sum of z-transformed measures) to one variable that was used in the regression analysis.

#### Mental age

Mental age (MA) was calculated using full scale IQ and chronological age (MA = CA x IQ/100). Full scale IQ was estimated with the Block design and Vocabulary subtests from Wechsler Intelligence Scale for Children-Fifth Edition (WISC-V) (Wechsler, 2014). These subtests were chosen due to their high reliability and a high correlation with the full scale IQ (Silverstein, 1983). Testing and scoring were done according to the manual. Block design is a subtest in which participants were asked to arrange a number of blocks according to a given pattern. Testing was stopped following two consecutive errors. Vocabulary is a subtest where the participants were asked to name pictures and describe the meaning of words. Testing was stopped following three consecutive errors.

#### Short-term and working memory

Two tests assessed verbal and visuospatial executive-loaded working memory; and three tests assessed visual, spatial, and phonological short-term memory respectively. Listening span, odd one out span, digit span and the Corsi blocks test are in the format of span tests which have three trials per list length, and the participants were allowed to continue to the next span level if two out of three trials were correct (both items and serial order). Item sequences start with one or two items, but become longer until the participant’s performance breaks down. The raw scores were the total number of trials correct. Listening span and odd one out span are measures of verbal and visuospatial executive-loaded working memory (ELWM). Listening span requires participants to listen to a sentence spoken by the examiner, state whether it is true or false, and then retain the last word of that sentence while subsequent sentences are presented and processed. Odd one out span requires participants to choose one out of three shapes that is different from the other two and shortly after retain its spatial position while subsequent odd one out decisions are made (Henry, 2001). Phonological short-term memory (PSTM) was measured with forward digit span from the WISC-V (Wechsler, 2014) and it requires participants to repeat a list of digits immediately in the same serial order as they were orally presented by the examiner. In order to ensure the same number of trials for all span tests, we added one extra trial at each list length to the forward digit span task using digits from the backward digit span assessment in the WISC-V. The Corsi blocks and visual sequential memory tests were used as measures of visuospatial short-term memory (VSSTM). The Corsi blocks test involves participants mimicking the examiner who taps a sequence of up to nine identical spatially separated blocks. Visual sequential memory, a subtest taken from Test of Visual Perception Skills Revised (TVPS-R) (Gardner, 1996), requires the participants to remember a sequence of shapes, and then shortly afterwards identify the correct sequence from a set of possibilities. The sequences are increased in length and testing is stopped following three consecutive errors. The raw score was the total number correct.

#### Rapid automatized naming

Rapid automatized naming (RAN) is a task that measures how quickly individuals can name aloud objects, colours, or symbols (letters or digits) (Wagner, Torgesen, & Rashotte, 1999). Participants were given two different RAN tests; they were asked to name as quickly as possible six different letters and to name six different colours, both presented randomly. Time in seconds was recorded. Note that this measure shows negative correlations in the analyses as shorter time indicates better performance. The raw score was the total number of seconds from both forms. Measures of both letters and colours were combined (sum of z-transformed measures) to one variable that was used in the regression analysis.

#### Home literacy

Home literacy was assessed using questionnaires for both parents and participants. Parents were asked about their first language and which language the family used in their home environment. This information was only used for inclusion decisions and to describe our group of participants. In addition, parents were asked about their completed educational level and reading habits. Completed educational level was scored on a four-point scale: grade 1–9 (1), grade 10–12 (2), university degrees (3), PhD education (4). Assessment of reading habits involved questions about how often the parents read different forms of literature (i.e. books, newspapers, comics, blogs/e-mails) how often they read for their child and how often they used to read for their child between the ages three and seven. Reading habits were scored on a four-point scale: never or almost never (1), 2–3 times a month (2), 2–3 times a week (3), everyday or almost everyday (4). The parents were asked to fill in the questionnaire at home and return it to the examiners.

The participants were asked about their reading habits and reading skills. Reading habits were measured with questions about how often the participants read different forms of literature (i.e. books, newspapers, comics, blogs/e-mails) and were scored on a four-point scale: never or almost never (1), 2–3 times a month (2), 2–3 times a week (3), everyday or almost everyday (4). In addition, they were asked how much they enjoy reading, measured on a four-point scale: not at all (1), a little (2), quite a lot (3), very much (4). The final question concerned how the participants rate their own reading abilities, measured on a four-point scale: poor (1), quite poor (2), quite good (3), good (4). The participants were asked all questions verbally during the assessment, and the questionnaire was filled in by the examiner. The raw score for home literacy was the total sum of scores from both parents and participants. For participants with only one parent answering the questionnaire, that parent’s score was doubled. Maximum score was 72.

#### Vision and hearing

Vision was screened using two different LEA-tests (Hyvärinen, Näsänen, & Laurinen, 1980); a 15-line distance chart (10 feet distance) and a near vision card (16 inches distance) to establish if the participants had normal vision. Participants with glasses were allowed to use them during testing. LEA-tests use symbols instead of letters or numbers. Participants with a visual acuity of 0.8 were included in the study. Hearing was screened with pure tone audiometry using a GSI 68 audiometer and an SA 201-IV audiometer and both were calibrated. The participants were wearing AudioCups during testing, to minimize impact from external noise. The participants were instructed to press a button every time they heard a tone. The screening process involved the following frequencies: 250, 500, 1000, 2000, 3000, 4000, 6000, and 8000Hz. Threshold was measured using standard audiological procedure and screening level was set to 20dB HL. Pure tone average (PTA) was calculated based on the following frequencies: 500, 1000, 2000, and 4000Hz. Participants with a PTA between 20–25dB HL were included in the study. For participants with hearing aids, pure tone audiometry is not applicable. However, these participants were included and coded as hearing aid users.

### Ethical approval

This study received ethical approval from the regional Research Ethics Committee in Linköping, Sweden (2017/139-31).

### Data analysis

All data analysis was done in R (R Core Team, 2017) and R packages. Missing values related to participants’ abilities (e.g., a participant not wanting to complete a specific test) were imputed with the minimum score of the other participants. Other missing values were treated as missing at random and values were imputed using the Multivariate Imputation by Chained Equations (MICE) approach, in the MICE package (van Buuren & Groothuis-Oudshoorn, 2011). Imputation of values was based on the other variables, but not the decoding variables. Descriptive and correlational data are presented for all variables. The four measures of decoding were entered into a principal component analysis (PCA) using the principal function in the psych package (Jolliffe, 2002). The main decoding variable was the PCA for a one component solution (nfactors=1, method=“regression”). We expected that the four decoding measures measure the same decoding construct, but this was tested by conducting a new PCA with Eigenvalue>1 as criterion. If there were to be more than one component, these rotated components would have been saved (rotate=“oblimin,” method=“regression”) and used in additional regression analyses. LASSO (Least Absolute Shrinkage Selector Operator) regression (Tibshirani, 1996) was conducted with the decoding variable as the dependent variable and the other 12 variables defined in the method section as independent variables. The advantage of LASSO compared to OLS regression is that LASSO has better prediction accuracy (inclusion of crossvalidation reduce overfitting) at the same time as it performs variable selection (Tibshirani, 1996). The tuning parameter lambda was chosen with the one standard deviation rule, this involves a compromise between optimum prediction accuracy (minimizes the cross-validation error curve) and interpretability (selection of fewer variables by moving one standard deviation in direction of increased regularization on the cross-validation error curve). LASSO has an assumption of linear relationships, which was checked. If the assumption had not been met, transformations of the problematic variables would have been made to meet the criterion.

The delay hypothesis was to be chosen if the predictor variables in the LASSO model were the same as in the hypothesis (i.e. RAN, phonological awareness, and (mental) age). Otherwise, the difference hypothesis would have been favoured. Interpretation was made of the included predictor variables and the remaining variables were considered to have limited practical relevance. The LASSO regression was done with the glmnet package (Friedman, Hastie, & Tibshirani, 2010). The packages papaja (Aust & Barth, 2020) and citr (Aust, 2016) was used for manuscript formatting, and tidyverse (Wickham, 2017) was used for data manipulation and the creation of plots.

## Results

The percentage of missing data was low for all variables (the maximum was 2.21% for any variable). The main decoding variable was calculated with a principal component analysis (PCA) on two measures of word recognition and two measures of phonological decoding. The PCA favored a one component solution with high loadings for all decoding measures (range 0.88 to 0.93) that explained 81.90% of the variance. For three assessments, composite measures were calculated by combining scores (verbal fluency 2 measures; RAN 2 measures; phonological awareness 3 measures). The sum of the z-transformed measures gave three composite variables used in the analysis. The intra-correlations between the measures ranged between 0.50 and 0.84.

Descriptive statistics of all included variables before transformation are provided in ***Table 1***.

### Correlations

***Table 2*** provides correlations for all variables included in the LASSO regression. All variables except for home literacy and vocabulary correlated significantly with decoding. Many predictor variables also correlated significantly with each other.

**Table 2:** Correlations between all variables included in the LASSO regression.


TEST	1	2	3	4	5	6	7	8	9	10	11	12	13

1 Decoding	1.00												

2 Phonological awareness	0.70	1.00											

3 RAN	–0.58	–0.35	1.00										

4 Verbal fluency	0.29	0.27	–0.40	1.00									

5 Vocabulary	0.11	0.24	–0.11	0.32	1.00								

6 Grammatical comprehension	0.27	0.41	–0.27	0.40	0.57	1.00							

7 Phonological STM	0.46	0.49	–0.23	0.19	0.11	0.27	1.00						

8 Spatial STM	0.21	0.18	–0.25	0.16	0.12	0.24	0.24	1.00					

9 Visual STM	0.33	0.39	–0.44	0.34	0.30	0.47	0.26	0.43	1.00				

10 Phonological ELWM	0.38	0.51	–0.29	0.35	0.29	0.50	0.41	0.08	0.35	1.00			

11 Visuospatial ELWM	0.23	0.28	–0.34	0.28	0.17	0.31	0.35	0.46	0.51	0.32	1.00		

12 Home literacy	0.01	–0.06	0.09	–0.04	0.03	–0.17	0.02	–0.12	–0.06	–0.02	–0.09	1.00	

13 Mental age	0.27	0.31	–0.37	0.30	0.44	0.45	0.20	0.22	0.43	0.25	0.42	0.02	1.00


### LASSO regression

**Figure 1:**
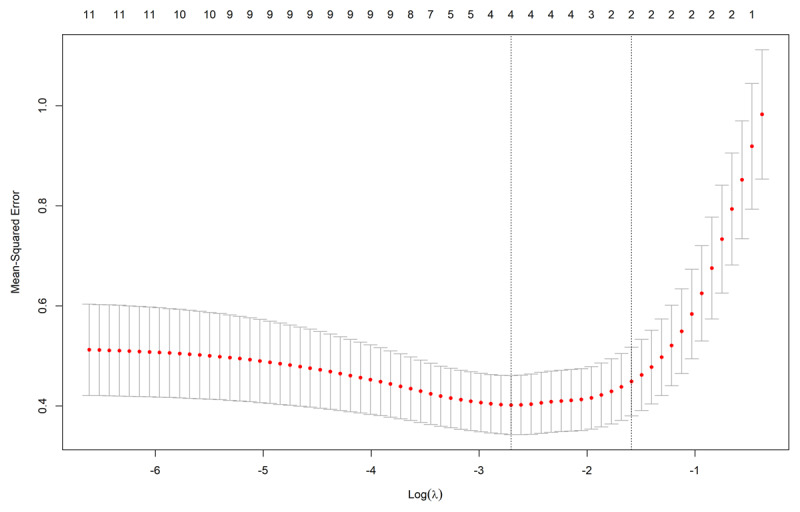
Mean-Square Error (MSE) as a function of lambda. The horizontal dotted line indicates the cross-validation curve. The vertical dotted lines indicate the two lambda values (left: lambda for minimal MSE, right: optimal lambda).

A LASSO regression was performed with decoding as the dependent variable and all other variables as predictor variables. The assumption of linear relationships was confirmed using visual inspection of scatterplots (available in supplements). The data was split in half forming a training dataset and a test dataset. First, the lasso model was fitted on the training dataset, and second, a cross-validation was performed on the training dataset. ***Figure 1*** shows a plot of training condition mean square error (MSE) as a function of lambda. From the cross-validation, the optimal lambda value (λ = 0.21) was chosen with the one standard deviation rule, see ***Figure 1*** for a visualization of how the MSE varies with lambda. Next, the optimal lambda value was applied to the test dataset.

**Figure 2:**
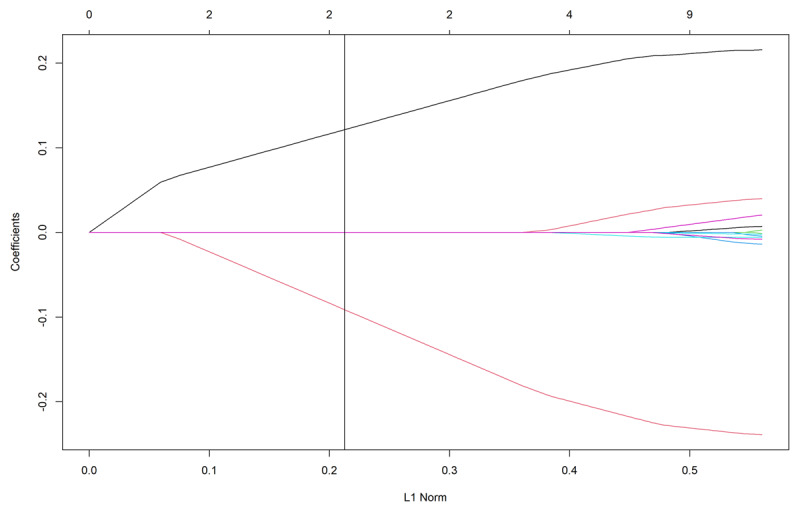
Visualization of coefficients from the LASSO regression. The y-axis indicates the coefficients, and the x-axis indicates the change of lambda value. The upper axis indicates the number of non-zero coefficients at the current lambda. The positive curve represents phonological awareness, and the negative curve represents RAN. The vertical line represents the optimal lambda value.

The LASSO regression selected two predictor variables of importance, phonological awareness (β = 0.16) and RAN (β = –0.15), all other variables were reduced to 0. ***Figure 2*** visualizes the coefficients, and each curve corresponds to a predictor variable. This clearly shows that phonological awareness (positive curve) and RAN (negative curve) emerge before the cut-off decided by the lambda value. This model explained 57.73% of the variance in decoding on the test data (64.49% on the training data). The results obtained from the LASSO regression support the delay hypothesis, since the two predictor variables of importance are the same as for the typically developing population.

### Exploratory analysis (not preregistered)

The finding that mental age was not selected in the LASSO regression analysis was not expected. Therefore, a first exploratory analysis was conducted by adding the two related variables chronological age and IQ as predictors of decoding. However, these predictors were not selected. A second exploratory analysis was conducted, where all participants with an estimated IQ of >70 were excluded. This analysis was conducted to ensure that the results were not driven by participants with an estimated IQ that was above the diagnostic threshold. In both exploratory analyses, the results were similar to the first LASSO regression (the same two predictors selected, and the same magnitude of the regression coefficients and the explained variance).

## Discussion

The present study investigated the concurrent predictors of decoding ability in a sample of adolescents with non-specific ID. The results showed that phonological awareness and RAN were the only variables related to decoding, hence supporting our delay hypothesis. The delay hypothesis refers to a pattern of relations between variables that are similar to findings in the literature on typically developing children, where RAN and phonological awareness are commonly reported as the variables of most importance in predicting decoding abilities (Hulme & Snowling, 2013; Landerl & Wimmer, 2008; Scarborough, 1998; Schatschneider, Fletcher, Francis, Carlson, & Foorman, 2004).

In the present study, phonological awareness had a stronger relationship with decoding than RAN, because it was the first variable picked by the LASSO regression. This is an interesting finding, since the opposite is often true for adolescent readers, especially in a transparent orthography. After the early years of schooling, phonological awareness diminishes as a predictor of decoding and decoding difficulties, while RAN persists as a predictor (Furnes & Samuelsson, 2010; Landerl & Wimmer, 2008). In our sample, phonological awareness seems to remain associated with decoding, suggesting that this group of readers has yet to undergo this developmental shift. This finding can be related to a large study by Ratz and Lenhard (2013), where the authors concluded that one third of individuals with ID (aged 6–21) decoded words letter by letter, which corresponds to the early alphabetic phases of decoding development in the typically developing population (Ehri, 2005). Together, these findings support a delayed profile of reading in individuals with non-specific ID.

As a part of the delay hypothesis, mental age was thought to be an important variable associated with decoding. In typically developing children, age is associated with decoding as years of schooling normally increases decoding performance, at least until decoding is fully mastered. However, this variable did not emerge as a contributor in our sample. To rule out that the variables composing mental age (chronological age and IQ) would have contributed with unique variance in the LASSO regression, these variables were entered separately in the regression in an exploratory analysis. These variables did not show an impact on decoding over and above phonological awareness and RAN.

In our sample, decoding was not fully mastered by the participants in terms of objective measures (norms from standardized tests), but they might have reached a reasonably stable level of decoding ability that was no longer affected by changes in mental and chronological age. In research on decoding ability in individuals with ID, many studies fail to report correlations between chronological age, mental age and other predictor variables and these age measures are rarely included in regression analyses. However, there are a few studies that have included these variables, and they show different results regarding the impact of chronological and mental age on decoding ability. In a study by Conners, Atwell, Rosenquist, and Sligh (2001), two groups with mixed aetiology ID divided by level of decoding performance (ages 8–12 years) differed significantly in chronological age. Another study, focusing on a sample with Down Syndrome aged 7–28 years and a mental age of 5–7 years, found that differences between the Down Syndrome group and the typically developing control group persisted even when mental age was accounted for (Verucci, Menghini, & Vicari, 2006). Furthermore, Henry and Winfield (2010) found that mental age was not related to single word reading in a group with non-specific ID aged 11–13 years, whereas this relationship was evident in the mental age matched control group. Thus, chronological age seems to be related to decoding ability in studies focusing on younger individuals with ID, suggesting that the ability to decode is still developing. However, in samples with a higher chronological age neither chronological age nor mental age seems to be associated with decoding ability, suggesting that the development of decoding abilities reaches a plateau. This plateau might in turn be a consequence of both cognitive and environmental factors. One possible explanation could be the lack of reading instruction focussing on phonics in later grades in Swedish special schools (Skolverket, 2018). Since the development of reading abilities occurs at a slower pace in individuals with ID (Allor, Mathes, Roberts, Cheatham, & Al Otaiba, 2014), it is reasonable to suggest that reading instruction targeting decoding should be part of the curriculum for an extended period of time.

Another possible explanation for the lack of an effect of mental age on decoding is that RAN and phonological awareness are both highly associated with mental age. Mental age had a stronger correlation with both RAN and phonological awareness compared to the correlation with decoding. This could indicate that the relationship between mental age and decoding is already accounted for by the variables chosen in the LASSO regression.

To conclude, the variables associated with decoding in a sample of adolescents with non-specific ID were similar to the variables identified in research on typically developing children. A large sample of adolescents with non-specific ID and an extensive set of different predictor variables increases confidence in the current findings. An important educational implication from these findings is that a phonemic awareness approach aimed at reinforcing accuracy and fluency in handling grapheme-phoneme correspondence could work in the same way, and to the same extent, in individuals with non-specific ID as for young typically developing children struggling to learn foundational skills of reading. Further research to support this suggestion is needed.

### Limitations

In this study we used two subtests from the WISC-V (Wechsler, 2014) to establish an estimate of IQ-level. This is common practice within research, but this method might have overestimated some participants’ IQ level as the participants had already been diagnosed with an intellectual disability partly through the use of intelligence tests. In addition, some participants’ IQ levels may have been underestimated, since the full-scale IQ is a combination of several tests and thus using only two tasks means there is the possibility for variance in performance between the different subtests. Thus, some caution might need to be taken over the interpretation of findings related to assessments of intelligence and mental age.

It is also important to consider the fact that adolescents who had non-specific ID and other co-occurring conditions such as ASD or ADHD were included in the current study. On the one hand, this decreased the internal validity and made it more difficult to draw firm conclusions about the impact of having a non-specific ID, as there may be effects of having both ASD/ADHD and ID that we were unable to unpick. On the other hand, this increased the external validity as our sample reflects the population we are trying to understand.

Two problems that might arise when conducting regression analyses are the selection of predictor variables and the risk of overfitting the model. Hence, we chose to use a LASSO regression to handle these problems with a robust method. Although this is the best practice, the selection of predictor variables can still be biased.

## Data accessibility statement

Raw data with guidance notes, analysis script, supplementary material, stage 1 IPA manuscript, and a laboratory log are available at: *https://osf.io/qdp5h/*.
